# Differential Proteomics Reveals miR-155 as a Novel Indicator of Liver and Spleen Pathology in the Symptomatic Niemann-Pick Disease, Type C1 Mouse Model

**DOI:** 10.3390/molecules24050994

**Published:** 2019-03-12

**Authors:** Melissa R. Pergande, Antony Cougnoux, Rathnayake A. C. Rathnayake, Forbes D. Porter, Stephanie M. Cologna

**Affiliations:** 1 Department of Chemistry, University of Illinois at Chicago, Chicago, IL 60607, USA; mperga2@uic.edu (M.R.P.); rrathn2@uic.edu (R.A.C.R.); 2 Division of Translational Medicine, Eunice Kennedy Shriver National Institute of Child Health and Human Development, Bethesda, MD 20879, USA; antony.cougnoux@nih.gov (A.C.); fdporter@mail.nih.gov (F.D.P.); 3 Laboratory for Integrative Neuroscience, University of Illinois at Chicago, Chicago, IL 60607, USA

**Keywords:** Niemann-Pick type C1, spleen proteomics, AJS-ESI mass spectrometry, lysosomal storage disorder, miR-155

## Abstract

Niemann-Pick disease, type C1 (NPC1) is a rare, autosomal recessive, lipid storage disorder caused by mutations in *NPC1*. As a result, there is accumulation of unesterified cholesterol and sphingolipids in the late endosomal/lysosomal system. Clinically, patients can present with splenomegaly and hepatomegaly. In the current study, we analyzed the differential proteome of the spleen in symptomatic *Npc1*^−/−^ mice to complement previous studies focused on the differential proteome of the liver, and then evaluated biomolecules that may serve as tissue biomarkers. The proteomic analysis revealed altered pathways in NPC1 representing different functional categories including heme synthesis, cellular regulation and phosphoinositide metabolism in both tissues. Differential proteins included several activators of the ubiquitous and critical protein, Akt, a major kinase involved in multiple cellular processes. Evaluation of Akt revealed decreased expression in both the liver and spleen tissues of symptomatic *Npc1*^−/−^ mice. Upstream regulation analysis also suggested that miR-155 may modulate the differences of known downstream protein targets observed in our dataset. Upon evaluation of miR-155, we observed an increased expression in the liver and decreased expression in the spleen of symptomatic *Npc1*^−/−^ mice. Here, we propose that miR-155 may be a novel indicator of spleen and liver pathology in NPC1.

## 1. Introduction

Niemann-Pick disease, type C1 (NPC1) is a rare, autosomal recessive, lipid storage disorder caused by mutations in *NPC1*, thereby resulting in intracellular lipid trafficking defects leading to the accumulation of unesterified cholesterol and sphingolipids in the late endosomal/lysosomal system [1]. This lipid accumulation causes both visceral and neurological defects which may manifest clinically as hepatosplenomegaly, neonatal jaundice, pulmonary failure or neurodegeneration (reviewed in References [2,3,4,5]). Currently, there is no FDA-approved therapy for NPC1, highlighting the need for a deeper understanding of altered biological pathways that may provide new therapeutic targets that can be used to monitor disease progression. The *Npc1* null mouse model, hereafter referred to as *Npc1*^−/−^ mice, recapitulates the human disease and presents with progressive cerebellar neurodegeneration, ataxia, hepatosplenomegaly and a shorted lifespan [6,7,8].

Although significant advances in understanding NPC1 pathogenesis have been reported, studies focusing on the spleen and liver have been limited. A subset of patients develop liver dysfunction, which is particularly relevant for the infantile form of the disease [9]. Remarkably, previous reports in the literature have indicated that between 45–65% of NPC patients present with neonatal cholestasis, where 10% die of liver failure prior to 6 months of age [10]. Hepatosplenomegaly is a well-documented symptom of lysosomal storage disorders, specifically NPC1 [11]. Enlargement of the liver can cause secondary enlargement of the spleen due to portal hypertension. Furthermore, early histology studies of *Npc1*^−/−^ mice reported characteristic histopathologic lesions in hepatocytes and reticuloendothelial cells of the spleen [12]. Zhang et al. evaluated the cellular components of the enlarged spleen in *Npc1*^−/−^ mice and reported an increase in the number of hematopoietic stem cells and macrophages [13]. In previous studies, we analyzed the differential proteome of the liver of the NPC1 mouse model at a symptomatic time point of disease progression and identified potential biomarkers [14]. In this study, we sought to analyze the differential proteome of the spleen to complement our previous proteomic analysis of the liver. With this information, we aimed to unravel the molecular mechanism leading to hepatosplenomegaly observed in *Npc1*^−/−^ mice.

Using differential proteomic technologies, we found new proteins which may serve as biomarkers for NPC1 spleen and liver pathology. Initially, we used mass spectrometry to perform label-free quantitative proteomics of spleen tissue in the NPC1 mouse model at a symptomatic time point of the disease. This was followed by pathway, functional and upstream regulator analysis of proteins with differential expression between genotypes of both spleen and liver proteomes. Notably, evaluation of late stage tissues implements a reverse engineering design which permits assessment of newly discovered biomarkers to monitor disease progression. Differential proteins included several activators of the ubiquitous and critical protein kinase, Akt. Related, performing upstream regulator analysis of the differential proteome indicated Tcl1a as a central connection among our data. Tcl1a enhances Akt phosphorylation which has been reported to be altered in NPC1 [15]. Finally, upstream regulator analysis of the differential proteomes also suggested that miR-155 may be a transcriptional regulator of specific altered proteins. Previous studies have shown that miR-155 is a negative regulator of Phosphatidylinositol 3,4,5-trisphosphate 5-phosphatase-1 expression [16,17]. The results of this study suggest that miR-155 may be a novel indicator of spleen and liver pathology in NPC1.

## 2. Results

### 2.1. Evaluation of the Differential Proteomes

**Figure 1:**
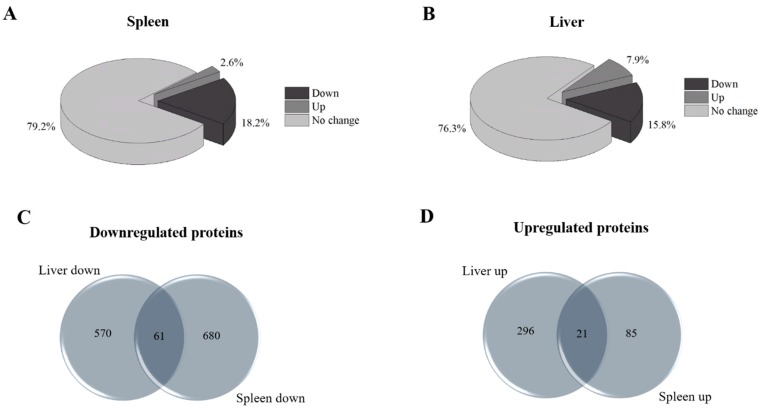
Comparative analysis of the differential proteome of the spleen and liver of 11-week *Npc1*^−/−^ mice. Differential proteins from the mass spectrometry analysis of the spleen (**A**) and liver (**B**) of *Npc1*^−/−^ mice where analyzed for downregulated and upregulated proteins. Evaluation of the proteome of the spleen revealed that 18.6% of the measured proteome was found to be downregulated while 2.6% was upregulated. The differential proteome of the liver was evaluated and revealed that 15.8% of the measured proteome was found to be downregulated while 7.9% was upregulated. Venn diagram analysis of the downregulated (**C**) and upregulated (**D**) proteins was performed to determine common altered proteins.

In this study, we evaluated the differential proteomes of both the spleen and liver of *Npc1*^−/−^ mice. Analysis of the spleen proteome via AJS-ESI mass spectrometry resulted in the identification of 39,852 ± 4637 peptides corresponding to 4082 ± 187 proteins (≥2 unique peptides per protein). Further analysis of the differential proteome of the spleen revealed 849 proteins that were altered in *Npc1*^−/−^ mice (≥2 fold and *p* ≤ 0.05) compared to control. All mass spectrometry results, including differential protein assignments, are provided in Table S2. Evaluation of the differential proteome of the spleen revealed that 18.6% of the measured proteins were downregulated while 2.6% were upregulated (Figure 1A). Given the proximity and complementary nature of the spleen and liver, we also evaluated the differential liver proteome of the same time point in a more extensive manner than our previous study [14]. Analysis of the liver revealed that 15.8% of the measured proteome was found to be downregulated while 7.9% was upregulated (Figure 1B). Venn diagram analysis for differential proteins in both tissues was overlapped to determine common alterations in each tissue (Table S3). Figure 1C revealed an overlap of 61 proteins that are downregulated in both tissues. Among the common downregulated proteins are delta-aminolevulinic acid dehydratase, oxygen-dependent coproporphyrinogen-III oxidase, ferrochelatase, uroporphyrin, nogen-III synthase, *N*-myc-interactor, mannose-binding protein A, Ran-binding protein 3 and exportin-7. Figure 1D revealed an overlap of 21 proteins that are upregulated in both tissues. Among these upregulated proteins are fatty acid binding protein-5, cathepsin D and macrophage-expressed gene 1 protein. By evaluating the differential proteome of both the liver and spleen, common proteins altered in major visceral organs in *Npc1*^−/−^ mice have been identified and provide insight into the pathology of peripheral NPC1 disease.

### 2.2. Pathway Analysis

**Figure 2:**
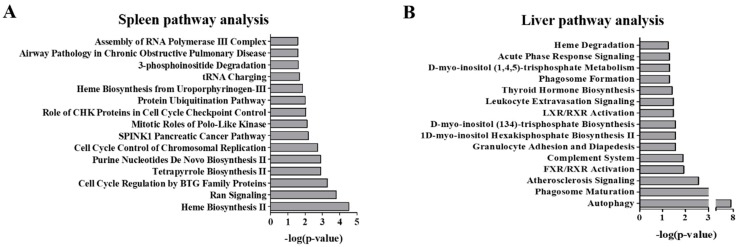
Pathway analysis of the differential proteome of the spleen and liver of 11-week *Npc1*^−/−^ mice. Differential proteins from the mass spectrometry analysis of the spleen (**A**) and liver (**B**) of *Npc1*^−/−^ mice were enriched for the top 15 pathways. Significance was determined using a Right-tailed Fisher’s exact test to determine probability of pathways from the Ingenuity Pathway Analysis Knowledge Base Library to those most significantly enriched.

Analysis of the differential proteome of the spleen revealed alterations in multiple pathways representing heme synthesis, cellular regulation and phosphoinositide synthesis and degradation (Figure 2A). These findings overlap the previous transcriptomic analysis of the spleen of *Npc1*^−/−^ mice [18]. Decreased expression of proteins in the spleen specific to heme biosynthesis include: uroporphyrinogen-III synthase, ferrochelatase, 5-aminolevulinate synthase, delta-aminolevulinic acid dehydratase, oxygen-dependent coproporphyrinogen-III oxidase and porphobilinogen deaminase. Related to this, decreased expression of hemogen was measured. Heme degradation was also one of the most significantly altered pathway in the differential liver proteome (Figure 2B, Table S3). Additionally, decreased expression of proteins specific to Ran signaling were also found in the spleen including importin subunit beta-1, exportin-2, Ran GTPase-activating protein-1, exportin-1 and importin subunit alpha-1. Further, manual inspection of mass spectrometry data revealed decreased expression of additional Ran signaling proteins including Ran binding protein-3 and multiple nuclear exportin and importin proteins: exportin-4, exportin-7, importin-5, importin-7, importin-11, importin subunit alpha-4 and importin alpha subunit-7 (Table S2). Interestingly, exportin-7 and Ran-binding protein-3 were also decreased in the liver of *Npc1*^−/−^ mice (Table S3).

Remarkably, we observe alterations of phosphatidylinositol regulatory proteins in both tissue types, however the contributing altered proteins were different for each tissue type. Here, phosphatidylinositol 5-phosphate 4-kinase type-2 alpha, phosphatidylinositol 4-kinase alpha, inositol 1,4,5-trisphosphate receptor type 3, inositol polyphosphate 1-phosphatase, inositol-3-phosphate synthase 1 and type 1 phosphatidylinositol 4,5-bisphosphate 4-phosphatase were observed to be altered in the liver. The following, phosphatidylinositol 4-kinase type 2-alpha, phosphatidylinositol 3,4,5-trisphosphate 5-phosphatase 1, 1-phosphatidylinositol 4,5-bisphosphate phosphodiesterase beta-1, 1-phosphatidylinositol 4,5-bisphosphate phosphodiesterase gamma-2 and type 2 phosphatidylinositol 4,5-bisphosphate 4-phosphatase levels were altered in the spleen (Table S3). Proteins related to autophagy were also modified in the spleen including cathepsin E and ubiquitin-like conjugating enzyme ATG3 which were downregulated while cathepsin D, cathepsin Z and cysteine protease ATG4B were upregulated. Markedly, autophagy was the top ranked altered pathway of the liver.

### 2.3. Functional Analysis

**Figure 3:**
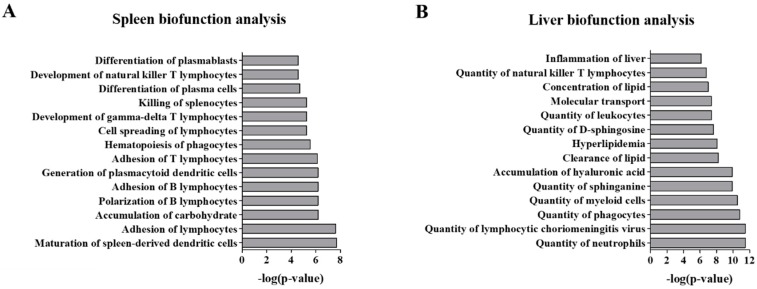
Functional analysis of the differential proteome of the spleen and liver of 11-week *Npc1*^−/−^ mice. Differential proteins from the mass spectrometry analysis of the spleen (**A**) and liver (**B**) of *Npc1*^−/−^ mice where enriched for top biological functions. Significance was determined using a Right-tailed Fisher’s exact test to determine probability of pathways from the Ingenuity Pathway Analysis Knowledge Base Library to those most significantly enriched.

Further analysis of the differential spleen and liver proteomes was performed to further elucidate functional alterations. Figure 3A shows the top ranked functional pathways modified in *Npc1*^−/−^ spleen. These are primarily related to immune function and include: Maturation of spleen-derived dendritic cells, Adhesion of lymphocytes, Polarization of B lymphocytes and Adhesion of B lymphocytes, Adhesion of lymphocytes and Cell spreading of lymphocytes. On the other hand, analysis of the differential liver proteome revealed functional alterations including: Quantity of leukocytes, neutrophils, myeloid cells, phagocytes as well as alterations of the Quantity of d-sphingosine and sphinganine (Figure 3B).

### 2.4. Alterations in Proteins Known to Be Transcriptionally Regulated by Tcl1a

**Figure 4:**
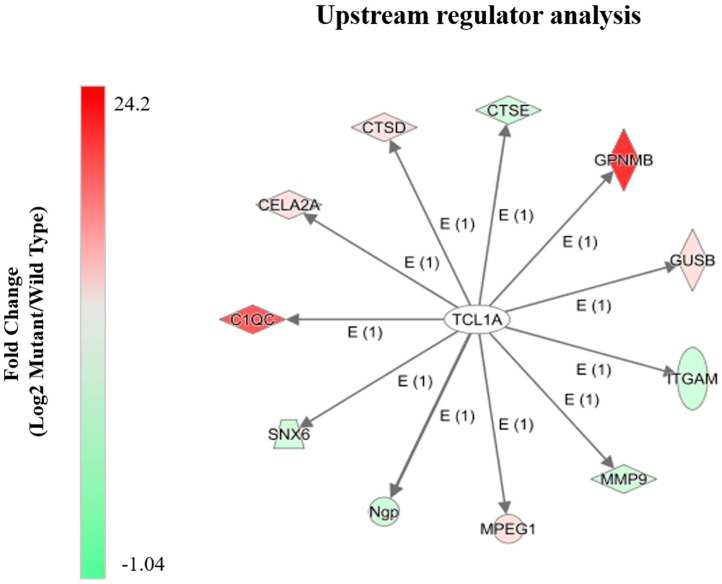
Upstream regulator analysis of the differential proteome of the spleen from 11-week *Npc1*^−/−^ mice. Predicted upstream regulator analysis from 11-week *Npc1*^−/−^ mice suggest that Tcl1a is a common regulator of various deferential proteins of the spleen. Assignment was based on the Ingenuity Pathway Analysis Knowledge Base Library. GPNMB = Transmembrane glycoprotein NMB, GUSB = Beta-glucuronidase, ITGAM = Integrin alpha-M, MMP9 = Matrix metalloproteinase-9, MPEG1 = Macrophage-expressed gene 1 protein, Ngp = Nucleolar GTP-binding protein 1, SNX6 = Sorting nexin = 6, C1QC = Complement C1q subcomponent subunit C, CELA2A = Chymotrypsin-like elastase family member 2A, CTSD = Cathepsin D and CTSE = Cathepsin E.

Upstream regulator analysis of the differential spleen proteome implicated T-cell leukemia/lymphoma protein 1a (Tcl1a) as a common transcriptional regulator of multiple proteins altered in our dataset and known to be involved in immune function (Figure 4). Tcl1a is highly abundant in the immune system, specifically in the spleen and plays a central role in lymphomagenesis [19]. Tcl1a also acts as a co-activator of the ubiquitous and critical, RAC-alpha serine/threonine-protein kinase (Akt) and as a signal enhancer to other interacting protein partners [20]. Interestingly, many of the pathways shown in Figure 2 are related to immune function. Transmembrane glycoprotein NMB is a negative regulator of T-cell activation and observed to act as a feedback regulator of proinflammatory response in macrophages [21]. Integrin alpha-M plays a pivotal role in modulating T-cell mobility, proliferation and differentiation [22]. Matrix metalloproteinase-9 has been reported to be important for cell migration [23]. Macrophage-expressed gene 1 protein is a macrophage-specific protein important for the maintenance of host defense [23], which is consistent with previous reports of increased macrophages in *Npc1*^−/−^ mice [13]. Neutrophilic granule protein has also been reported to be a negative regulator of lymphangiogenesis and tumor metastasis [24]. Complement C1q subcomponent subunit C has been reported to be essential for development of host innate immune response [25]. Importantly, previous studies involving complement in *Npc1*^−/−^ mice reported that it is not required to mediate neurodegeneration and subsequent neuroinflammation [26]. Together, these data suggest that Tcl1a may be a repressor of various proteins of the spleen of *Npc1*^−/−^ mice that are important for activation and migration of immune cells.

### 2.5. Altered Expression of Akt in the Spleen and Liver

**Figure 5:**
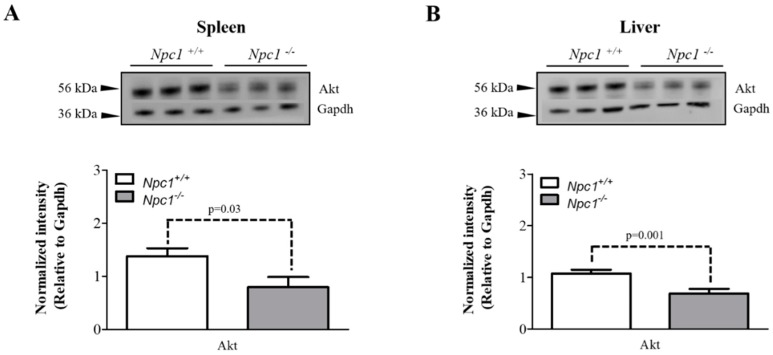
Western blot analysis of Akt in spleen and liver tissue of 11-week *Npc1^+/+^* and *Npc1*^−/−^ mice. Protein lysates from the spleen and liver of 11-week *Npc1^+/+^* and *Npc1*^−/−^ mice (N = 3 each genotype) were subject to electrophoresis and Western blotting. Data analysis revealed decreased expression of Akt in both the (**A**) spleen and (**B**) liver of *Npc1*^−/−^ mice. Data is reported as relative to Gapdh.

Given that Tcl1a is a known co-activator of Akt, we sought to evaluate total Akt protein expression levels in the spleen of 11-week *Npc1^+/+^* and *Npc1*^−/−^ mice. Western blot analysis revealed significantly decreased expression of Akt in *Npc1*^−/−^ compared to *Npc1^+/+^* mice (Figure 5A). While Akt was identified in our current study, the differential analysis did not result in a statistically significant difference. We owe this finding to the fact that in label-free approaches, mostly large changes are typically observed. In our previous studies, we evaluated the differential liver proteome of 11-week *Npc1*^−/−^ mice and observed alterations in pathways involved in downstream signaling of Akt [14] and these findings are consistent with previous microarray data [18,27]. With this connection and the decrease observed in the spleen, we sought to evaluate the expression of Akt in the liver of 11-week *Npc1^+/+^* and *Npc1*^−/−^ mice. Western blot analysis revealed significantly decreased expression of total Akt in liver tissue from *Npc1*^−/−^ compared to *Npc1^+/+^* mice (Figure 5B). Therefore, we report decreased expression of total Akt in both the spleen and liver of *Npc1* null mice at end stage of the disease. Given the wide number of downstream signaling pathways regulated by Akt, additional studies are needed to determine the functional relationship between the observed decreases in the phosphoinositide regulatory enzymes and Akt observed in *Npc1*^−/−^ mice. Additionally, phosphorylation of Akt is an important regulatory component for signaling and evidence of decreased pAkt in liver in NPC1 has been shown [15], further supporting our current findings.

### 2.6. miR-155 May Be a Marker of NPC1 Spleen and Liver Pathology

**Figure 6:**
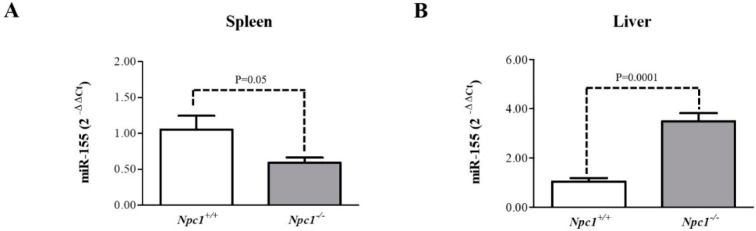
miR-155 analysis of spleen and liver tissue from 9-week *Npc1^+/+^* and *Npc1*^−/−^ mice. miR-155 was selected for evaluation by quantitative real-time PCR in spleen and liver tissue from *Npc1^+/+^* and *Npc1*^−/−^ mice (N = 5 for each genotype). The levels of miR-155 were significantly decreased in the (**A**) spleen and increased in the (**B**) liver of *Npc1*^−/−^ mice. The expression level of the miR-155 in each tissue was normalized to endogenous U6 snRNA.

Upstream transcriptional regulator analysis of the differential spleen proteome also implicated micro RNA-155 (miR-155) as a possible modulator of altered biological processes found in this study. miR-155 is multifunctional with previous studies suggesting one role as a positive regulator of T-cell mediated immune response via the production of inflammatory cytokines [28]. Additionally, miR-155 has been noted to regulate the expression of DnaJ heat shock protein family (Hsp40) member A2 [29] and Phosphatidylinositol 3,4,5-trisphosphate 5-phosphatase 1 [16,17], both proteins which were observed to be decreased in our mass spectrometry analysis (Table S2). With this insight, we measured the relative expression of miR-155 in the liver and spleen of 9-week *Npc1^+/+^* and *Npc1*^−/−^ mice, a slightly earlier time point than the initial proteomic studies. Figure 6A reveals a decreased expression of miR-155 in the spleen of *Npc1*^−/−^ compared to *Npc1^+/+^* mice. Interestingly, miR-155 is involved in the regulation of multiple genes involved in lipid metabolism. Lin et al. observed that expression of miR-155 in the liver of transgenic mice was able to induce a general downward trend in the expression profile of hepatic genes involving lipid metabolism [30]. Specifically, microarray data from the study mentioned above reported decreases in *Evolv5* and *Fads2*, consistent with previous studies in *Npc1*^−/−^ mice [27]. Furthermore, our previous studies of the NPC1 mouse model revealed alterations in the liver proteome specific to lipid metabolism, including fatty acid and triglyceride synthesis [14], which is also consistent with other studies on *Npc1*^−/−^ mice [18,27]. Thus, we also evaluated the relative expression of miR-155 in liver tissue of 9-week *Npc1*^−/−^ and *Npc1^+/+^* mice. Figure 6B reveals a significant increase in the expression of miR-155 in the liver of *Npc1*^−/−^ mice. Together these data suggest that miR-155 may be involved in the pathophysiology of NPC1 and may play a role in regulating the expression of various spleen and liver proteins.

## 3. Discussion

In the current study, we used capillary LC-MS/MS to perform label-free quantitative proteomics and analyzed the differential proteome of the spleen of *Npc1*^−/−^ mice at an end-point in disease progression. These data revealed unique alterations of the proteome and furthermore, when analyzed in conjunction with the differential liver proteome provided insight into alterations in NPC1. Our results from the analysis of the spleen were overlapped with results from our previous study of the liver proteome at the same time point of disease progression. Analysis of the datasets revealed changes in pathways related to heme synthesis, immunity, phosphoinositide metabolism and autophagy in both the liver and spleen. We also evaluated the expression of total Akt in both tissues and observed a decrease in both the spleen and liver of *Npc1*^−/−^ mice. In addition, evaluation of miR-155 was observed to be decreased in the spleen and increased in the liver of *Npc1*^−/−^ mice. With this, we propose that miR-155 may be a novel monitor of spleen and liver pathology in NPC1.

The differential proteome of *Npc1* mutant spleen tissue revealed decreased expression of all enzymes specific for the synthesis of heme. In addition, alterations in multiple enzymes involved in this pathway were also observed in the liver, although more pronounced in the spleen. It is important to note that the amount of free intracellular heme is the main rate-limiting step in this pathway [31]. Additionally, cells can employ a longer lasting control over expression via regulation of transcription and translational mRNA 5-aminolevulinic acid synthase through the formation of heme-DNA adducts [32,33]. Moreover, reduced heme synthesis can result in cytochrome deficiency, specifically heme can induce mRNA synthesis of cytochrome P-450. In previous studies, investigation of the P450 system in *Npc1*^−/−^ mice revealed significant changes in the gene expression of many P450 associated genes across the entire lifespan of the mice [34]. Alteration in these cytochrome enzymes can result in the generation of reactive oxygen species [35]. Similar alterations in enzymes involved in heme biosynthesis have been reported in liver tissue suggesting impairment of lymphocytes in patients with malignant lymphoproliferative disorders [35]. Markedly, we observed a decrease in hemogen in the spleen, a protein responsible for the proliferation and differentiation of hematopoietic cells, potentially a result of the availability of free heme. Further, our data revealed alterations of specific proteins related to the activation and function of macrophages in both tissues, supporting the previously reported increase in the number of activated macrophages in the tissues [18]. Additional experiments would be needed to determine whether the alterations of heme degradation enzymes are directly related to the impairment and migration of immune cells in visceral NPC1 organs. In general, the findings of this study suggest possible alterations in both the liver and spleen in the feedback mechanisms that regulate the synthesis of heme, a molecule needed for the proper function of proteins such as hemoglobin. Additional studies measuring the amount of intracellular heme concomitant to the expression of heme regulated mRNAs would be needed in order to confirm this hypothesis.

Proteomic analysis in the spleen and liver also implicated alterations in ras-related nuclear (Ran) signaling. This signal transduction pathway is essential for cell cycle progression, nuclear envelope structure and function and translocation of RNA and proteins though nuclear pores. Cluzeau et al. performed microarray analysis in the liver across the lifespan of *Npc1*^−/−^ mice and observed decreased expression of both Ran Guanine nucleotide exchange factor and Ran GTPase activating protein supporting our proteomic data and suggesting the regulation of this pathway occurs at the transcription level [27]. Indeed, studies regarding Ran signaling and immune function have been reported. Nieland et al. observed that lymphoma cells transfected with Ran become less tumorigenic [36]. Sekimoto et al. observed STAT-1 translocation to nuclei depends on Ran and its GTPase activity [37]. Notably, STATs are essential for immune function [38]. Suzuki et al. also observed an increase of STATs in NPC1 with consecutive activation of the toll-like receptor-4 pathway [39]. Here, we also observed decreased expression of Stat1, Stat3, Stat5A and Stat5B in the spleen. Thus, we propose that the altered expression of Ran signaling proteins may result in cellular trafficking defects that could impair functions related to immunity in *Npc1*^−/−^ mice.

We also observed a significant decrease of total Akt in both liver and spleen of *Npc1*^−/−^ mice. Akt is a serine/threonine kinase that is critical in signal transduction pathways involved in cell proliferation, apoptosis, angiogenesis and diabetes. Each of the three isoforms (Akt1, Akt2 and Akt3) display a high degree of sequence homology but differ slightly in the localization of their regulatory phosphorylation sites and expression distribution. Studies in NPC1 patient fibroblasts have reported decreased phosphorylation of Akt [40]. Analysis of liver transcriptome revealed decreased expression of Akt2 in *Npc1*^−/−^ mice [27]. Therefore, with reasonable inference, we believe that the major form of Akt detected in this experiment is Akt2, however further studies evaluating the expression of each isoform would be needed to confirm this assumption. Members of the Tcl1 family have been reported to bind to the PH domain of Akt to enhance the substrates phosphorylation [41]. Interestingly, Du et al. reported that activation of the Akt/mTOR pathway increased NPC1 protein degradation by ∼50% [42]. Additional studies would be needed to evaluate whether alterations in Tcl1a are directly responsible for the decreased phosphorylation of Akt observed in NPC1. The results of this study suggest that therapeutic intervention stimulating the activity of the Akt signaling pathway may prove beneficial in the treatment of NPC1; however, overexpression studies monitoring Akt signaling members in parallel with the measurement of lysosomal cholesterol and disease phenotype would be needed to support this hypothesis. On the other hand, the targeting of specific pathways regulated by Akt may pose a challenge as it is the central signaling member to multiple downstream pathways.

Upstream transcriptional regulator analysis suggested that miR-155 may be functionally related to NPC1 pathophysiology. We measured the expression of miR-155 in *Npc1^+/+^* and *Npc1*^−/−^ tissues and observed decreased expression in the spleen and increased expression in the liver of *Npc1^-/-^* mice. miR-155 is a multifunctional miRNA that is highly expressed in activated B and T-cells and macrophages and also plays a role in various physiological and pathological processes such as hematopoiesis and immunity [43,44]. Deregulation of miR-155 has been reported to be highly expressed in certain cancers, specifically those malignancies of B cell or myeloid origin and transgenic over-expression of miR-155 in mice results in cancer [43,45]. This micro RNA is also increased in Parkinson disease and implicated in neurodegeneration [46]. In a screening of miRNAs in NPC1 fibroblasts, miR-155 was found to exhibit decreased expression [47]. In this study, we observed tissue specific alterations. Therefore, these data suggest that alterations in miR-155 may be cell type specific, supporting the divergence between our spleen and liver results. More recently, overexpression of miR-155 in the liver of transgenic mice was observed to alter the expression profile of hepatic genes associated with lipid metabolism [30]. To the best of our knowledge, this is the first report of altered expression of miR-155 in *Npc1*^−/−^ mice. Further evaluation of altered miR-155 expression would be needed to determine whether the decreased expression in the spleen is related to the activation of immune cells and increased expression in the liver related to liver failure in NPC1.

## 4. Materials and Methods

### 4.1. Reagents and Chemicals

All reagents were acquired from Sigma-Aldrich (St. Louis, MO, USA) and used as received unless otherwise noted.

### 4.2. Animal Breeding and Tissue Collection

All animal experiments were performed according to NICHD, NIH and UIC institutional animal care committee- approved protocols (Approved numbers: NICHD 18-002 and UIC 18-163). *Npc1^+/+^* and *Npc1*^−/−^ mice (BALB/cNctr-*Npc1^m1N/m1N^*, Jackson Laboratories) were euthanized via CO_2_ asphyxiation at 9-weeks (N = 5 for each genotype) and 11-weeks of age (N = 3 for each genotype) and spleen and liver tissues collected, flash frozen on dry ice and stored at −80 °C until analysis.

### 4.3. Sample Preparation for Proteomic Analysis

Samples were thawed on ice and protein lysates made for each of the 11-week control and mutant tissues. Cells were lysed in RIPA buffer (50 mM Tris pH 8, 150 mM NaCl, 1% Triton-X 100, 0.5% sodium deoxycholate, 0.1% sodium dodecyl sulfate, complete EDTA-free proteinase inhibitor cocktail (Roche, Basel, Switzerland)) and protein concentration was determined by BCA assay (Pierce-Thermo, San Jose, CA, USA). Control and mutant lysate pools were made by combining equal amount of protein from each animal (1.2 mg total for each tissue pool). Next, a recombinant green fluorescent protein (GFP) internal standard was spiked in at a concentration of 100 fmol per 1 µg protein extract prior to sample preparation via the filter-aided sample preparation (FASP) as previously described [48,49]. Briefly, proteins where diluted in 8 M urea, reduced with 5 mM dithiothreitol (DTT) for 20 min at 55 °C, alkylated with 20 mM iodoacetamide at room temperature for 20 min and buffer exchanged with 50 mM ammonium bicarbonate. Trypsin was added (1:25 *wt*/*wt*) and proteins digested overnight at 37 °C. Peptides were eluted with 80 μL of 50 mM ammonium bicarbonate followed by a final 100 μL rinse of the filter with 100 mM sodium chloride. Samples were desalted and concentrated using an HLB cartridge (Waters Corporation, Milford, MA, USA). The resulting digest for each genotype was fractionated into 20 samples using a Waters XBridge BEH130 C18 3.5 μm 4.6 × 250 mm column using a Lab Alliance HPLC system as previously described [50]. All samples were dried and re-suspended in 45 µL of 0.1% (*v*/*v*) formic acid prior to mass spectrometry analysis.

### 4.4. LC-MS/MS Analysis

Proteomic analysis was performed via label-free quantitative mass spectrometry using an Agilent 6550 iFunnel Q-TOF LC/MS system controlled by Agilent Mass Hunter acquisition software. The mass spectrometer was operated in 2 GHz extended dynamic range mode, data dependent acquisition performed in positive ion mode and internal reference mass calibration *m*/*z* 922.0098 was used. Ten microliters of each fraction were loaded onto a 2.1 × 100 mm Poroshell 120 EC-18, 2.7 µm column (Agilent Technologies Inc., Santa Clara, CA, USA). Mobile phase A was 0.1% formic acid and mobile phase B was acetonitrile. Peptide separation was performed using the following gradient: 3% B at 0–5 min, 30% B at 45 min, 60% B at 50 min, 90% B at 53 min, hold at 90% B until 60 min at a flow rate of 200 µL/min. Acquisition parameters for mass spectrometry analysis were as follows: 10 MS scans/second (*m*/*z* 300–1700), 3 MS/MS scans/second (*m*/*z* 50–1700) and maximum precursor selection of the top 20 ions of charge states ≥2 with collision energy maintained with slope = 3 and offset = 2 for +2 charge state species and slope = 3.6 and offset = −4.8 for species with charge state ≥3. Source parameters were as follows: drying gass temperature (250 °C), drying gas flow (14 L/min), nebulizer (35 psi), sheath gas temperature (250 °C), sheath gas flow (11 L/min), VCap (3,500 V) and Oct 1 RF Vpp (750 V). Analysis of each fraction was performed in triplicate and all raw mass spectrometry data was deposited and is publicly available at ftp://massive.ucsd.edu/MSV000083201.

### 4.5. Protein Identification and Relative Quantification

LC-MS/MS data was analyzed using Spectrum Mill Version B.06.00.201 HF1 (Agilent Technologies Inc., Santa Clara, CA, USA) against the UniprotKB/SwissProt database (2017), which contained 16,935 sequences for *Mus musculus*. An in-house GFP database was created in Spectrum Mill and incorporated into database searches. All search parameters were as follows: precursor mass tolerance was 20 ppm and fragment mass tolerance was 50 ppm, enzyme was trypsin with two missed cleavages and carbamidomethylation (C) as a fixed modification and oxidation (M) and deamidation (NQ) as variable modifications. Auto thresholds were used for peptide identification to achieve a target 1% false discovery rate. Search results from Spectrum Mill were imported into Mass Profiler Professional (Agilent Technologies Inc., Santa Clara, CA, USA) for label-free quantitation using the median peptide abundance for each protein. Here, the intensity for all identified GFP proteins was plotted and reviewed (Figure S1), where it was determined that normalization between replicates was not needed. Retention times of eluting peptides from comparative samples were aligned prior to analysis. Only peptides with charges from +2 to +4 containing two or more isotopic peaks were included and proteins were further filtered to those containing a minimum of 2 unique peptides prior to relative quantification.

### 4.6. Differential Proteome Analysis

Following mass spectrometry analysis, we sought to evaluate common proteomic-based alterations in the liver and spleen in NPC1. First, we compared the spleen and liver differential proteomes (fold change ≥ 2 and *p* ≤ 0.05) using Venn diagram analysis using the Mass Profiler Professional software. It is important to note that the differential liver proteome data came from our previous results [14]. Next, pathway and functional analysis were performed using the differential proteins from both datasets using the Ingenuity Pathway Analysis software (IPA, Ingenuity Systems, Redwood City, CA, USA). Here, significance was determined using a Right-tailed Fisher’s exact test to calculate a *p*-value to determine probability of pathways and biofunctions from the IPA Knowledge Base to those most significantly enriched. Lastly, upstream transcriptional regulator analysis was used to predict transcriptional regulators for the differential proteome of the spleen and liver.

### 4.7. Western Blot Analysis

The RIPA generated protein lysates for each of the 11-week animals were subject to electrophoresis and Western blotting. Here, the samples were denatured in Laemmli buffer containing 50 mM DTT at 70 °C for 10 min before loading onto a 12% NuPage Bis-Tris gel. Proteins were separated by SDS-PAGE (120 V for ~90 min) and transferred to a nitrocellulose membrane via a Western blotting apparatus (Novex Life Technologies, Carlsbad, CA, USA) at 12 V for 45 min. Membranes were blocked in a solution of 5% (*wt*/*v*) nonfat dry milk and 0.1% (*v*/*v*) Tween-20 (PBS-T) overnight before addition of primary antibody (anti-Akt/PKB clone SKB1, 1:1000 dilution, Millipore) followed by the addition of HRP conjugated secondary antibody (anti-mouse IgG 1: 2500 dilution) in 5% (*wt*/*v*) milk PBS-T for one hour. Excess secondary antibody was removed by washing with PBS-T three times before protein bands were visualized by incubation with Pico West Blotting Substrate (Thermo Pierce Biotechnology, Rockford, IL, USA) and imaging on an Azure c300 imaging system (Dublin, CA, USA). Western blot signals were compared via densitometry analysis using ImageJ (v1.52a) [51]. GraphPad Prism (v5.01) (San Diego, CA, USA) was used to determine statistical significance using an unpaired *t*-test.

### 4.8. RNA Isolation and qRT-PCR Analysis

Total RNA was extracted from the spleen and liver tissue of the 9-week control and mutant mice with TRIzol reagent and 1 µg of each sample reversely transcribed with SuperScript III (Thermo Fisher Scientific Waltham, MA, USA) as previously described [30,52]. Quantitative RT-PCR was done using a Bio-Rad CFX qRT-PCR system (Biorad, Foster City, CA, USA) with Fast SYBR Green Master Mix (Thermo Pierce Biotechnology, Rockford, IL, USA). All primers (Integrated DNA Technologies Skokie, IL, USA) were used as received. Transcript levels of miR-155 and U6 snRNA (endogenous control) were carried out as previously described [30]. The sequences for each primer can be found in Table S1. Each SYBR green reaction was performed with 4 µL cDNA, 10 µL Fast SYBR Green Master Mix, 0.4 µM forward primer, 0.4 µM reverse primer and water to adjust the final volume to 20 µL. All the reaction mixtures were incubated in a 96-well plate at 95 °C for 2 min, followed by 40 cycles of 94 °C for 15 s and 60 °C for 1 min. miR-155 expression levels were calculated (2^−ΔΔ^*^C^*^t^) via the Livak method [53]. Statistical significance between groups was determined using an unpaired *t*-test in the GraphPad Prism software.

## 5. Conclusions

This study reports alterations of biomolecules in the spleen and liver at a late stage of NPC1 that may be indicators of visceral organ pathology. Analysis of the proteomes from the spleen and liver of *Npc1^-/-^* mice revealed alterations in pathways related to heme synthesis, cellular regulation, phosphoinositide metabolism and autophagy in both tissues. Evaluation of total Akt revealed decreased expression in both the liver and spleen. Additionally, we observed decreased expression of miR-155 in the spleen and conversely, increased expression in the liver of *Npc1*^−/−^ mice.

## Supplementary Materials

Supplementary Materials are available online. Figure S1: GFP peptide intensities; Table S1: Primers used for miRNA analysis; Table S2: Mass spectrometry analysis of spleen proteomics; Table S3: Venn diagram results; Table S4: Pathway analysis results; Table S5: Function analysis results.
