# Supplementary material for: Differential Proteomics Reveals miR-155 as a Novel Indicator of Liver and Spleen Pathology in the Symptomatic Niemann-Pick Disease, Type C1 Mouse Model
Source: Molecules. 2019 Mar 12;24(5):994. doi: 10.3390/molecules24050994 (PMC6429457; doi:10.3390/molecules24050994)
Supplement: Supplementary file 1 [file molecules-24-00994-s001.zip › molecules-448744-SI/Supporting Information Molecules.pdf]

miR-155 is a novel indicator of liver and spleen pathology in the symptomatic Niemann-Pick disease, type C1 mouse model

Melissa R. Pergande[1], Antony Cougnoux[2], Rathnayake A. C. Rathnayake[1], Forbes D. Porter[2] and Stephanie M. Cologna[1,3]

Affiliations:

1. Department of Chemistry, University of Illinois at Chicago, Chicago, IL 60607, USA
2. Division of Translational Medicine, *Eunice Kennedy Shriver* National Institute of Child Health and Human Development, Bethesda, MD 20879, USA.
3. Laboratory for Integrative Neuroscience, University of Illinois at Chicago, Chicago, IL 60607, USA

Keywords: Niemann-Pick, type C1, spleen proteomics, AJS-ESI mass spectrometry, lysosomal storage disorder, miR-155

Correspondence:

Stephanie M. Cologna, Ph.D.
Assistant Professor
Department of Chemistry and Laboratory for Integrative Neuroscience
University of Illinois at Chicago
P: 312-413-2762
E: cologna@uic.edu

Primers for qRT-PCR analysis of microRNAs

Primer name	Primer sequence
U6 snRNA-RT	5'-AACGCTTCACGAATTTGCGT-3'
U6 snRNA-Forward primer	5'-CTCGCTTCGGCAGCACA-3'
U6 snRNA-Reverse primer	5'-AACGCTTCACGAATTTGCGT-3'
MiR155-RT primer	5'-GTCGTATCCAGTGCAGGGTCCGAGGTATTCGCACTGGATACGACACCCC-3'
MiR155-Forward primer	5'-GACTGTTAATGCTAATCGTGATAG-3'
MiR155-Reverse primer	5'-GTGCAGGGTCCGAGGTATTC-3'

Table S1: Primers used for microRNA analysis.

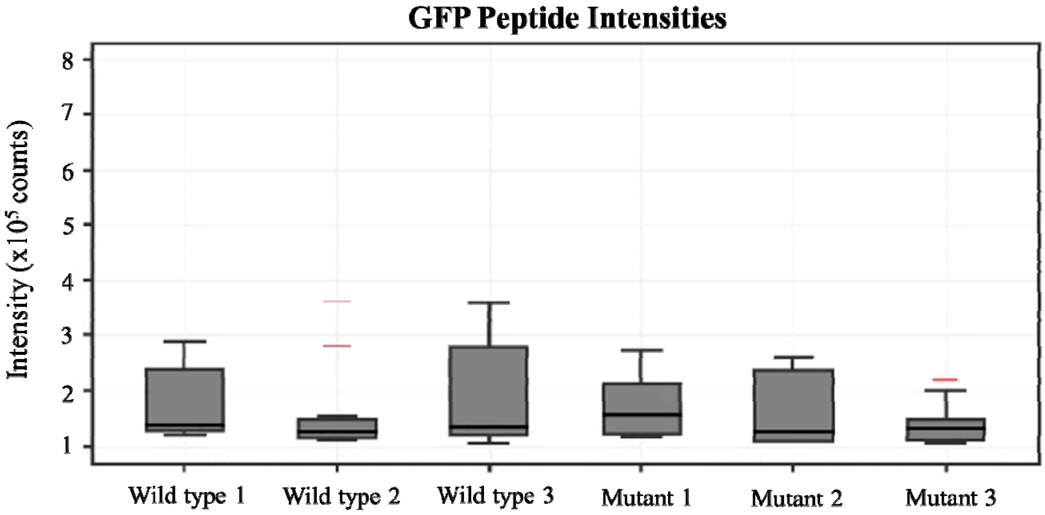

Figure S1. Intensity of peptides measured for the internal standard (GFP protein). LC-MS/MS data for each of the wild type and mutant technical replicates was reviewed in order to determine whether intensity normalization was needed between replicates. Here, the intensity for the measured GFP peptides were plotted and it was determined that normalization between samples was not needed as the median intensity was comparable between all replicates.
